# The relationship of serum visfatin levels with clinical parameters, flow-mediated dilation, and carotid intima-media thickness in patients with ankylosing spondylitis

**DOI:** 10.3906/sag-2012-351

**Published:** 2021-08-30

**Authors:** Rabia AYDOĞAN BAYKARA, Adem KÜÇÜK, Ayça TUZCU, Göksel TUZCU, Erkan CÜRE, Ali Uğur USLU, Ahmet OMMA

**Affiliations:** 1 Department of Physical Medicine and Rehabilitation, Malatya Training and Research Hospital, Turgut Özal University, Malatya Turkey; 2 Department of Rheumatology, Faculty of Medicine, Necmettin Erbakan University, Konya Turkey; 3 Department of Biochemistry, Faculty of Medicine, Adnan Menderes University, Aydın Turkey; 4 Department of Radiology, Aydın Ataturk State Hospital, Aydın Turkey; 5 Department of Internal Medicine, Ota & Jinemed Hospital, İstanbul Turkey; 6 Department of Internal Medicine, Yunus Emre State Hospital, Eskişehir Turkey; 7 Department of Rheumatology, Ankara Numune Training and Research Hospital, Ankara Turkey

**Keywords:** Visfatin, ankylosing spondylitis, flow-mediated dilation, carotid intima-media thickness, subclinical atherosclerosis

## Abstract

**Background/aim:**

Atherosclerotic heart diseases can occur at an early age in patients with ankylosing spondylitis (AS). Flow-mediated dilation (FMD) and carotid intima-media thickness (cIMT) values are reliable markers for early detection of subclinical atherosclerosis in patients with AS. We aimed to investigate the relationship between visfatin levels and indirect markers of subclinical atherosclerosis and endothelial dysfunction in patients with AS.

**Materials and methods:**

Forty-two patients diagnosed with AS and 42 age, sex, and body mass index (BMI)-matched controls were included in the study. Visfatin levels, FMD, and cIMT were measured using appropriate methods.

**Results:**

Visfatin levels of the patients were significantly higher than controls (p < 0.001). FMD values in patients with AS were significantly lower (p = 0.007) whereas cIMT were significantly higher than the controls (p = 0.003). There was a negative relationship between FMD with visfatin levels (p = 0.004), BASDAI (p = 0.010), and BASFI (p = 0.007). There was a positive relationship between cIMT with visfatin (p = 0.005), BASDAI (p < 0.001), and BASFI (p < 0.001). There was a positive relationship between visfatin with BASDAI (p < 0.001), and BASFI (p < 0.001).

**Conclusion:**

Visfatin levels are increased and associated with impaired FMD and increased cIMT in patients with AS. Increased visfatin levels may be associated with subclinical atherosclerosis in AS.

## 1. Introduction

Ankylosing spondylitis (AS) is a chronic inflammatory disease-causing destruction in the spinal and peripheral joints [1]. Multilevel organ and system involvement including eye, skin, kidney, gastrointestinal, and cardiovascular systems may occur in the course of AS. Cardiovascular system involvement is seen in 2% to 10% of patients with AS and cardiovascular risk is increased compared to the healthy population [2–4]. Cardiac involvement may occur in various forms ranging from asymptomatic atherosclerosis to mortal conduction disorders, ischemic heart disease, aortic valve diseases, aortitis, hypertension, and cardiomyopathy [5]. Findings of atherosclerosis may be detected even at an early stage of the disease, and chronic inflammation is considered as an important contributing factor in the development of atherosclerosis in AS [6].

The carotid intima-media thickness (cIMT) measurement is suggested as a cost-effective, reliable, and noninvasive method for detecting subclinical atherosclerosis in patients with AS [7]. Flow-mediated dilatation (FMD) is another noninvasive method for early detection of endothelial dysfunction which reflects the dilation rate of an artery due to nitric oxide released from endothelial cells [8]. FMD can detect endothelial dysfunction caused by decreased bioavailability of nitric oxide released from the endothelium [9]. Impaired FMD can be detected before apparent atherosclerotic changes may occur and is an early and reliable marker of endothelial damage [10].

Visfatin also called nicotinamide phosphoribosyltransferase (NAMPT) and pre-B cell colony enhancing factor (PBEF), was first described in 2004[11]. Visfatin is an adipokine predominantly released from visceral adipose tissue but also released from all tissues [12].Visfatin is a pro-inflammatory cytokine and also increases the release of other pro-inflammatory cytokines such as interleukin (IL) -1beta, IL-6, and tumor necrosis factor-alpha from monocytes and vascular endothelial cells and results in severe inflammation [13]. Increased visfatin levels are associated with vascular inflammation and carotid plaques [14]. Increased circulatory visfatin levels have been reported in various inflammatory diseases such as rheumatoid arthritis, inflammatory bowel diseases, and psoriasis [12]. Elevated visfatin levels has been reported as a predictor of radiographic progression in patients with AS [15,16]. Visfatin levels were associated with increased cIMT values and impaired FMD [17,18].

In this study, we aimed to investigate the possible relationship between endothelial dysfunction and visfatin levels in patients with AS.

## 2. Materials and methods

### 2.1. Patients 

Forty-two patients with AS who met the modified New York criteria and 42 age-, sex-, and body mass index (BMI)-matched healthy controls were included in the study. Patients were consequently recruited from Malatya State Hospital rheumatology and physical medicine and rehabilitation outpatient clinics. Individuals who were pregnant or nursing women, or had diagnosis for malign tumors, diabetes mellitus, hypertension, heart disease, hyperlipidemia, acute or chronic infections, acute or chronic renal failure, chronic obstructive pulmonary disease, and obesity (BMI ≥ 30 kg/m^2^) were excluded. 

#### 2.1.1. Sample size

The prevalence of AS is 0.5%–1.4%. The incidence of atherosclerosis in the community is 27.6% [19]. Atherosclerosis is seen 1.4–1.7-fold more in AS patients than in the population [20]. The minimum sample size required to find statistical significance was calculated with the Sample Size Calculator Sample Size Calculator [clincalc.com])., considering 0.05 type I error (alpha), 0.8 power (1-beta), effect size 0.68, and the two-sided alternative hypothesis (H1). The minimum number of patients and controls to be included in the study was calculated as 36 each. 

#### 2.1.2. Current smoker

Current smokers were recorded according to the definition of the National Health Interview Survey “Current smoker: An adult who has smoked 100 cigarettes in his or her lifetime and who currently smokes cigarettes.” The smoking duration was calculated as packs-year^1^https://www.smokingpackyears.com/.. 

### 2.2. Biochemical analysis

Venous blood samples of the patients and controls were collected after 12 h of fasting. Blood samples were taken into dry tubes, divided and separated into small pieces, and stored at –80 °C until analyzed. Complete blood count analysis was performed by flow cytometry device (Mindray BC-6800 Auto Hematology Analyzer, Shenzhen, China). C-reactive protein (CRP), aspartate aminotransferase (AST), alanine aminotransferase (ALT), blood urea nitrogen (BUN) creatinine values ​were measured using a spectrophotometry device (Abbot-Architect c8000, Japan). The erythrocyte sedimentation rate (ESR) was evaluated by the Westergren method (Berkhun SDM-100, Turkey).

### 2.3. Visfatin measurement

Serum blood samples for visfatin were taken from all patients and controls between 08.00–09.00 in the morning after 12 h of fasting. Visfatin measurement of all individuals was made from their serum samples on the same day. Serum visfatin level was measured by enzyme-linked immunosorbent method (ELISA) using the Elisa kit (Elabscience, China). The process was carried out following the manufacturer’s instructions. Absorbance was evaluated at 450 nm by an ELISA reader.

### 2.4. Ultrasonography

Ultrasonographic (US) measurements were made by the same experienced radiologist using a high-resolution ultrasound device (Logiq S6; General Electric, Milwaukee, WI, USA) and a 12 MHz multi-frequency linear probe. Sonography was performed by the patient in the supine position and the neck turned to the side of examination. US evaluations in patients and controls were performed in the morning (9:00–10:00 AM) after 12 h of fasting. Twelve hours before the study, all subjects were discontinued smoking, alcohol, and caffeine consumption, and exercise are not permitted.

### 2.5. Measurement of carotid intima-media thickness

Intima-media thickness (IMT) measurements were attained from nonplaque areas of the three different points of the right and left common carotid artery (CCA) and 1 cm distant from the bifurcation. Two bright echogenic lines on the arterial wall were identified as intima and media. A total of three measurements were made for each side of the body, the average of three measurements was calculated as the IMT value. 

### 2.6. Method of evaluating flow-mediated dilation

The participants were placed in the supine position and rested for at least 10 min. Then, the brachial blood pressure was measured and noted. Subsequently, FMD was measured using an echography (Vivid S6, GE, USA) equipped with a linear probe (frequency range, 12 MHz). The basal measurement of the right brachial artery diameter was performed in a linear plane and nearly 2 to 3 cm upper from the antecubital fossa. Afterward, a cuff was placed around the forearm distal to the ultrasonographic evaluation line. The cuff was inflated to supra systolic pressure (50 mm Hg above the previously measured systolic blood pressure) and held in this position for 5 min of ischemia. The diameter of the brachial artery was re-measured 1 min after the cuff was completely deflated. FMD value was obtained by calculating the percentage increase in diameter of the brachial artery. 

### 2.7. Statistical analysis

SPSS program (version 20) was used for the evaluation of all statistical analyzes in the study. Kolmogorov–Smirnov test was used to show the homogeneity of distribution. The t-test or Mann–Whitney U test was used for comparison between groups and Pearson correlation coefficients or Spearman rank test for analyzing relationship between parameters where appropriate. The categorical variables such as age and current smoking were evaluated using the chi-square test. Independent variables affecting cIMT and FMD were determined using linear stepwise regression analysis. Before performing stepwise linear regression analysis, univariate analysis was performed to determine independent variables associated with cIMT and FMD. In univariate analysis, for cIMT, age, male sex, visfatin, Bath ankylosing spondylitis disease activity index (BASDAI), Bath ankylosing spondylitis functional index (BASFI), fasting plasma glucose (FPG), total cholesterol (TC), low-density cholesterol (LDL), triglyceride (TG), CRP, and ESR were determined as independent variables. For FMD, BASDAI, BASFI, disease duration, creatinine, TC, and LDL were determined as independent variables. A p-value of < 0.05 was considered statistically significant. 

## 3. Results

Age, sex, and BMI values of the patients and controls were similar (p > 0.05). Alcohol and tobacco consumptions were similar between groups. The regions where we detect enthesitis in patients are: costochondral joints (n = 4), trochanter major (n = 3), anterior superior iliac spine (n = 3), iliac crest (n = 1), posterior superior iliac spine (n = 1), processus spinosus (n = 6), achilles tendon region (n = 4), and multiple sites (n = 2). The median disease duration of patients with AS was 3.5 years. Sociodemographic characteristics of the patients and healthy controls are shown in Table 1.

**Table 1:** Sociodemographic characteristics of the patient and control groups.

	AS(n = 42)	Control(n = 42)	P value
Age (years)	39.2 ± 7.3	39.4 ± 9.6	0.929
Sex (M/F) (n)	13/29	13/29	1.000
Disease duration (years)	3.5 (1.0–45.0)		
Peripheral arthritis n, (%)	2 (4.8)		
Enthesitis n, (%)	24 (57.1)		
BASDAI	3.2 ± 1.3		
BASFI	2.8 ± 1.3		
BMI (kg/m2)	26.8 ± 3.6	25.9 ± 5.0	0.361
Current smokers (n)	13	16	0.412
Smoking (packet-years)	18.5 ± 4.3	14.6 ± 8.0	0.108
Drinking (n)	1	0	1.000
NSAID (n)	21		
MTX (n)	14		
Infliximab (n)	5		
Adalimumab (n)	4		
Etanercept (n)	6		
Certolizumab (n)	4		
Salazopyrin (n)	2		
Topical steroid	1		
Systemic steroid	0		

Abbreviations: AS, ankylosing spondylitis; BASDAI, bath ankylosing spondylitis disease activity index; BASFI, bath ankylosing spondylitis functional index; BMI, body mass index; NSAID, nonsteroidal antiinflammatory drug; MTX, methotrexate.

**Figure 1:**
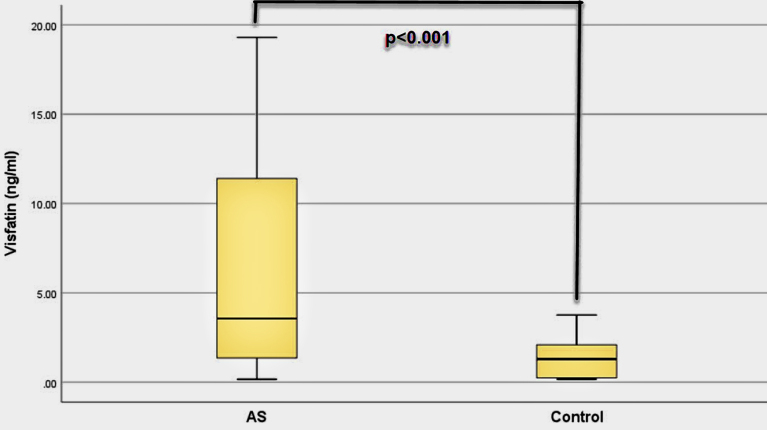


**Figure 2:**
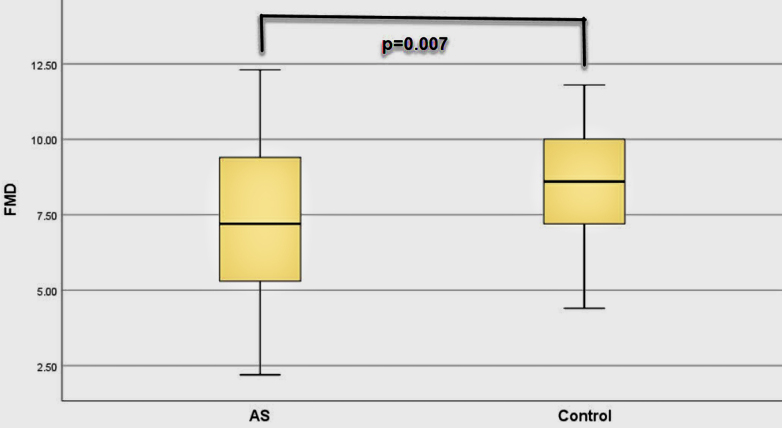


**Figure 3:**
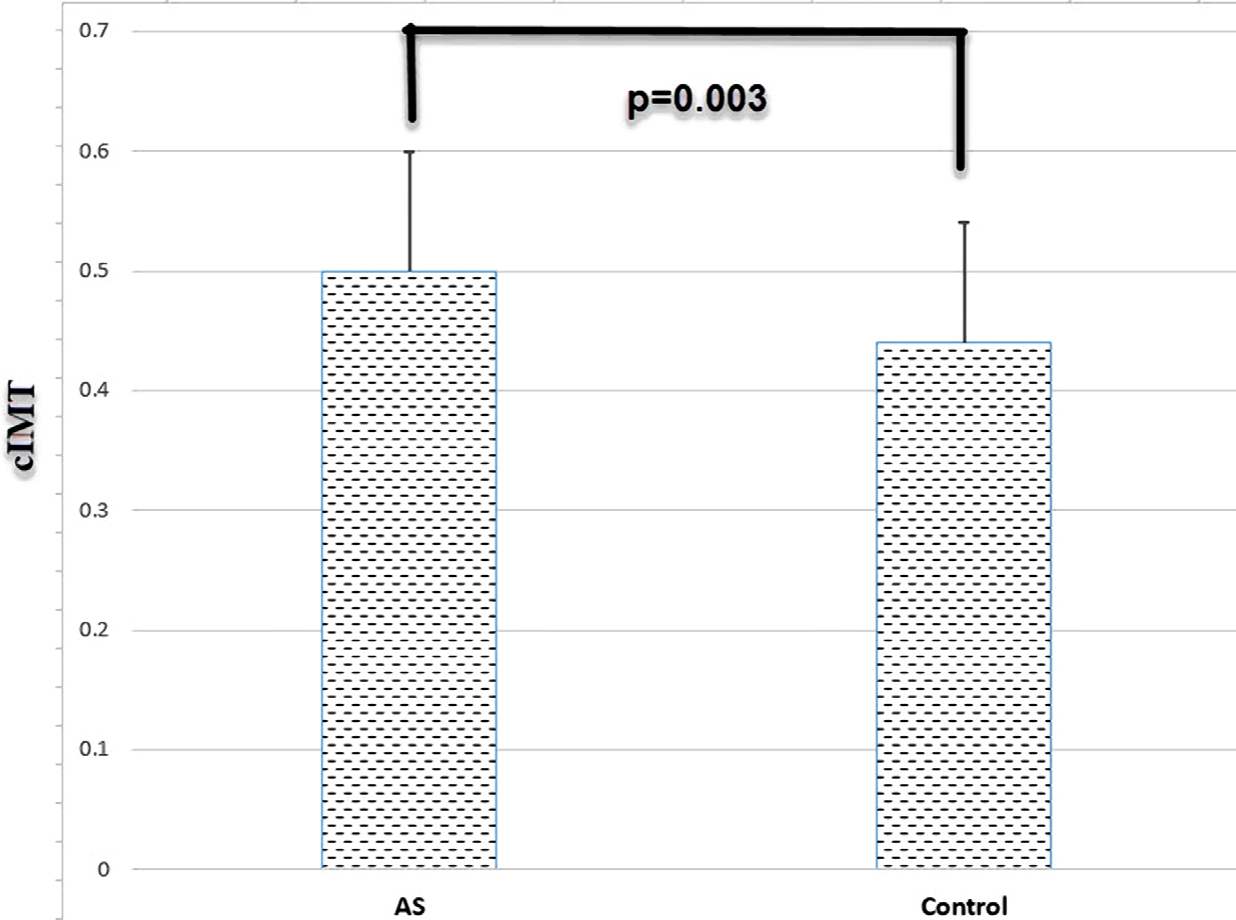


Visfatin levels of the patients were significantly higher than healthy controls (p < 0.001) (Table 2). FMD values of the patients were lower than controls (p = 0.007), whereas their cIMT values were higher than the controls (p = 0.003). Serum uric acid, CRP and ESR values of the patients were higher than the controls (Table 2). High-density lipoprotein (HDL) values of the patients were lower than healthy controls. Visfatin, FMD, and cIMT values of the patients and controls are shown in Figures 1, 2, and 3, respectively. All biochemical results of the patients are shown in Table 2.

**Table 2:** Biochemical results of the patient and control group.

	AS (n = 42)	Control (n = 42)	P value
Visfatin (ng/mL)	3.5 (0.19–19.3)	1.3 (0.17–7.0)	<0.001
FMD (%)	7.2 ± 2.8	8.7 ± 1.7	0.007
cIMT mm	0.50 ± 0.1	0.44 ± 0.1	0.003
Carotid plaque (n)	0	0	1.000
FPG (mg/dL)	99.6 ± 22.3	93.4 ± 16.1	0.144
BUN (mg/dL)	25.8 ± 7.5	29.9 ± 8.2	0.020
Creatinine (mg/dL)	0.7 ± 0.08	0.7 ± 0.18	0.106
AST (IU/L)	22.0 ± 11.9	22.8 ± 10.2	0.733
ALT (IU/L)	27.4 ± 18.7	24.8 ± 16.3	0.502
SUA (mg/dL)	4.3 ± 0.9	3.3 ± 0.6	<0.001
CRP (mg/dL)	0.20 (0.10–4.82)	0.10 (0.10–2.15)	0.008
ESR (mm/h)	23.2 ± 17.5	14.7 ± 11.3	0.007
WBC (×109/L)	7.5 ± 2.0	7.2 ± 1.4	0.463
Hb (g/dL)	13.5 ± 1.9	13.3 ± 1.3	0.668
TSH (mIU/L)	1.7 ± 0.9	1.8 ± 1.1	0.788
TC (mg/dL)	191.3 ± 39.7	199.8 ± 25.2	0.250
TG (mg/dL)	131.9 ± 52.5	128.8 ± 53.7	0.792
HDL (mg/dL)	40.2 ± 8.6	48.3 ± 9.8	<0.001
LDL (mg/dL)	124.7 ± 35.6	125.7 ± 19.8	0.883

Abbreviations: AS, ankylosing spondylitis; FMD, flow-mediated dilation; cIMT, carotid intima-media thickness; FPG, fasting plasma glucose; BUN, blood urea nitrogen; AST, aspartate aminotransferase; ALT, alanine aminotransferase; SUA, serum uric acid; CRP, C-reactive protein; ESR, erythrocyte sedimentation rate; WBC, white blood cell count; Hb, hemoglobin; TSH, thyroid stimulating hormone; TC, total cholesterol; TG, triglyceride; HDL, high-density lipoprotein; LDL, low-density lipoprotein.

There was a negative correlation between FMD values with visfatin (p = 0.004), BASDAI (p = 0.010), and BASFI (p = 0.007). There was positive relationship between cIMT with visfatin (p = 0.005), BASDAI (p < 0.001), and BASFI (p < 0.001). There was a negative relationship between cIMT and HDL. There was a positive relationship between visfatin with BASDAI (p < 0.001), and BASFI (p < 0.001). There was a negative relationship between visfatin and HDL. All results of correlation analysis are shown in Table 3.

**Table 3:** Correlation analysis results of patients.

Parameters	FMD		cIMT		Visfatin	
	R value	P value	R value	P value	R value	P value
Visfatin	–0310	0.004	0.301	0.005		
BASDAI	–0.281	0.010	0.396	<0.001	0.479	<0.001
BASFI	–0.294	0.007	0.427	<0.001	0.441	<0.001
SUA	–0.280	0.010			0.257	0.018
Age			0.506	<0.001		
FPG			0.219	0.045		
CRP			0.314	0.004		
ESR			0.229	0.036	0.236	0.030
Disease duration					0.368	<0.001
TC	–0.222	0.041	0.342	0.001		
HDL			–0.218	0.036	–0.229	0.036
LDL			0.322	0.003		
TG			0.407	<0.001		

Stepwise linear regression analysis was performed after finding independent variables related to cIMT and FMD according to univariate analysis. In the stepwise linear regression analysis (r^2 ^= 0.551, F = 24.2, p < 0.001 for cIMT; r^2 ^= 0.251, F: 6.6, p < 0.001 for FMD); there was an independent inverse relationship between FMD with visfatin (beta [β] = 0.223, p = 0.045), and BASFI (β = 0.290, p = 0.011). There was an independent association between cIMT with age (β = 0.431, p < 0.001), BASFI (β = 0.371, p < 0.001), and male sex (β = 0.298, p < 0.001). All results of univariate and stepwise linear regression analysis are shown in Table 4 and Table 5. 

**Table 4:** Independent variables associated with cIMT and FMF in univariate analysis.

	cIMT		FMD	
	Beta	P value	Beta	P value
Age	0.506	<0.001	0.028	0.799
Male sex	0.255	0.019	0.140	0.203
Visfatin	0.301	0.005	0.310	0.004
BMI	0.111	0.314	0.021	0.847
BASDAI	0.396	<0.001	0.281	0.010
BASFI	0.427	<0.001	0.294	0.007
FPG	0.219	0.045	0.041	0.713
BUN	0.069	0.533	0.013	0.909
Creatinine	0.020	0.854	0.281	0.010
TC	0.342	0.001	0.222	0.042
HDL	0.212	0.053	0.107	0.302
LDL	0.322	0.003	0.280	0.010
TG	0.407	<0.001	0.060	0.585
SUA	0.104	0.344	0.198	0.070
CRP	0.314	0.004	0.007	0.949
ESR	0.229	0.036	0.082	0.457
AST	0.101	0.359	0.139	0.208
ALT	0.184	0.094	0.184	0.094
WBC	0.022	0.845	0.132	0.232
Hb	0.054	0.623	0.014	0.896
TSH	0.040	0.715	0.062	0.574
Smoking	0.096	0.546	0.250	0.111
Disease duration	0.245	0.117	0.346	0.025

Abbreviations: cIMT, carotid intima-media thickness; FMD, flow-mediated dilation; BMI, body mass index; BASDAI, bath ankylosing spondylitis disease activity index; BASFI, bath ankylosing spondylitis functional index; FPG, fasting plasma glucose; BUN, blood urea nitrogen; TC, total cholesterol; HDL, high-density lipoprotein; LDL, low-density lipoprotein; TG, triglyceride; SUA, serum uric acid; CRP, C-reactive protein; ESR, erythrocyte sedimentation rate; AST, aspartate aminotransferase; ALT, alanine aminotransferase; WBC, white blood cell count; Hb, hemoglobin; TSH, thyroid stimulating hormone.

**Table 5:** Stepwise linear regression analysis.

Dependent variable	Independent variables	Beta regression coefficient	P value
FMD	Visfatin	–0.223	0.045
	Creatinine	–0.263	0.010
	BASFI	–0.290	0.011
	LDL	–0.219	0.031
cIMT	Age	0.431	<0.001
	BASFI	0.371	<0.001
	Male sex	0.298	<0.001
	TG	0.242	0.003

Abbreviations: FMD, flow-mediated dilation; cIMT, carotid intima-media thickness; BASFI, bath ankylosing spondylitis functional index; LDL, low-density lipoprotein; TG, triglyceride.

## 4. Discussion

Our results revealed that cIMT, a marker of subclinical atherosclerosis, was higher in patients with AS than matched healthy controls, and FMD, a marker of endothelial dysfunction, is lower in patients than controls, indication poorer endothelial functions. Our results also showed that serum levels of visfatin were higher in patients with AS compared to controls, and higher levels of visfatin may be associated with higher disease activity and poorer physical functions. 

The increased risk of atherosclerosis is known in patients with AS. Chronic inflammation which disrupts endothelial functions and steroid and nonsteroid antiinflammatory drugs used in the treatment are factors contributing to the increased cardiovascular risk in patients with AS [3,21,22].

Visfatin is a multiple immunomodulatory protein that stimulates the release of pro-inflammatory cytokines. Visfatin activates leukocytes and causes pro-inflammatory cytokine release, resulting in an increase in inflammation and reactive oxygen species [23]. Increased visfatin levels were shown associated with insulin resistance and increased cardiac events[24]. An independent relationship was found between visfatin levels with coronary artery disease and coronary slow-flow phenomenon[25]. Zheng et al. found a strong relationship between increased visfatin levels (>8.799 ng/mL) and major adverse cardiovascular events (MACEs) in acute myocardial infarction [26]. Stejskal et al. reported that the 20 ng/mL cut-off value of visfatin is an independent marker for AMI with high sensitivity (84%) and specificity (90%)[27]. Miranda–Filloy et al. found visfatin levels higher in AS patients than healthy controls, but they did not find any relationship between visfatin level with lipid parameters and BASDAI[28]. Hulejova et al. found a positive relationship between visfatin and BASDAI in patients with axial spondyloarthritis[28]. In the current study, we found a relationship between serum visfatin levels with both BASDAI and BASFI. We found a negative relationship between visfatin with HDL, which has a potent antiatherosclerotic effect.

Atherosclerosis and cardiac events can be seen at an early age in AS[29]. Impaired FMD is a good marker of subclinical atherosclerosis. Bodnar et al. found that FMD values are significantly lower and cIMT values remarkably higher in patients with AS compared to controls[30]. Wang et al. found that in 120 AS patients, the FMD value was pronouncedly lower than the control group[31]. There is an inverse relationship between the circulating visfatin levels with FMD, an early marker of endothelial dysfunction[32]. Yilmaz et al. showed a relationship between endothelial dysfunction and visfatin in 406 patients with chronic renal failure[33]. 

The cIMT value has been proven to be a reliable marker for early detection of subclinical atherosclerosis in patients with AS [7,34].A positive correlation was shown between serum visfatin levels and cIMT in diabetic and nondiabetic hemodialysis patients[35]. Zhong et al. reported that the relationship between serum visfatin levels and carotid plaque, and an increase in visfatin levels was a predictive marker for the carotid plaque with 70% sensitivity and 67% specificity[36]. 

Regarding rheumatic diseases which progress with aberrant inflammation, higher visfatin levels with respect to controls and high disease activity compatible with visfatin levels were shown in patients with rheumatoid arthritis and Behçet’s disease but no relationship between cIMT and glucose intolerance[37]. On the other hand, contradictory results were reported in patients with systemic lupus erythematosus and systemic sclerosis indicating similar levels of visfatin compared with controls[35]. Syrbe et al. reported higher serum visfatin levels in patients with AS[15].

To the best of our knowledge, our study is the first to investigate cIMT and FMD together and possible interactions between circulating visfatin levels and disease parameters in patients with AS. 

Possible interactions between visfatin and FMD with uric acid levels and estimated glomerular filtration rate (eGFR) has been described in different disease groups [38,39].In our study, we found a relationship between visfatin and FMD and serum creatinine. Low-density lipoprotein (LDL) is a highly proatherogenic molecule and Matsui et al., revealed a strong relationship between LDL and impaired FMD in statin naive individuals[40]. In our study, we also found a relationship between LDL and FMD.

## 5. Limitation of the study

Our study has some limitations. The study was conducted with a small number of subjects. Current study is a pilot study and studies with broad participation are needed. Another missing point in our study is the absence of the diseased-control group. The number of patients with high disease activity (BASDAI> 4) was quite low. The relationship between visfatin and cIMT and FMD in AS patients should be investigated in further studies.

## 6. Conclusion

Circulating visfatin levels are associated with disease activity and functional ability in patients with AS, and along with cIMT and FMD may be associated with increased risk of subclinical atherosclerosis and endothelial dysfunction.

## Funding

All authors declare that this study has received no financial support. 

## Informed consent

The study was approved by the ethics committee of Ankara Numune Education and Research Hospital, Turkey. All participants were informed of the study protocol and signed consents were obtained.
